# Adaptation and Validation of the Eudaimonic Well-Being Questionnaire to the Spanish Sport Context

**DOI:** 10.3390/ijerph18073609

**Published:** 2021-03-31

**Authors:** Rubén Trigueros, José M. Pérez-Jiménez, Alejandro García-Mas, José M. Aguilar-Parra, José M. Fernandez-Batanero, Antonio Luque de la Rosa, Ana Manzano-León, Noelia Navarro

**Affiliations:** 1 Hum-878 Research Team, Health Research Centre, Department of Psychology, University of Almería, 04120 Almería, Spain; 9josepj@gmail.com (J.M.P.-J.); aml570@ual.es (A.M.-L.); nng777@ual.es (N.N.); 2 GICAFE Research Group, Faculty of Psychology, University of the Balearic Islands, 07122 Palma, Spain; alex.garcia@uib.es; 3 Faculty of Education Science, Teaching and Educational Organization, University of Seville, 41004 Seville, Spain; batanero@us.es; 4 Department of Education, University of Almería, 04120 Almería, Spain; aluque@ual.es

**Keywords:** sport, eudemonic, well-being, psychometric properties, confirmatory factorial analysis

## Abstract

Studies to date that have focused on the well-being of the athlete have been based on the hedonic point of view. However, there is a second point of view: eudemonia. Therefore, the present study aims to validate and adapt the Eudemonic Well-Being Scale to the sport context. The study involved 2487 from several sport clubs. Several confirmatory factor analyses were carried out and showed that the six-factor questionnaire was the one with the best fit indices. These results show that the scale is in relation to the original scale (from Spain) and to Waterman’s theoretical model.

## 1. Introduction

In recent years, a multitude of studies are emerging in the field of sport psychology that focus on the ways in which athletes seek personal satisfaction [1,2]. This personal satisfaction of the athlete has commonly been understood from a hedonic point of view, focused on maximizing subjective happiness, enjoyment and pleasure, and minimizing subjective discomfort, strain and pain [2,3]. However, there is a second viewpoint that refers to eudemonia, which focuses on the pursuit of personal growth, excellence and virtue. In this sense, eudemonia is not just about seeking pleasant feelings, but rather about developing one’s capabilities and doing one’s best [4,5]. Different studies in the educational context have shown that it is key in the reinforcement of adaptive behaviors that favor academic performance and subjective wellbeing [6].

The eudemonic perspective views an individual’s subjective well-being as a state of positive psychological functioning, which is the result of engagement in one’s own activity, self-reflection and the pursuit of the best version of oneself through successive challenges [7,8]. However, authors such as Waterman argue that the individual’s experience of a state of eudemonia cannot be separated from hedonism. Despite this high correlation, hedonism and eudemonia show a number of differences [9]. In this sense, eudemonia activities require constant effort on the part of the individual, characterized by a balance between challenge and personal skills [10]. In contrast, hedonic activities require no personal effort and are related to positive emotions (e.g., happy, joyful, relaxed) [11]. Thus, hedonics arises from getting things one wants, while eudemonia is found among the things one wants. However, there are currently no known scales that assess eudemonia in the context of sport that could help to analyze the well-being of athletes in depth.

Currently, among the instruments available to measure eudemonic well-being, the Eudemonic Well-being Questionnaire designed by Waterman et al. [12], which is based on the theoretical postulates on eudemonia (see [9]) and which has been widely used in various studies [13,14,15,16], stands out. This instrument was designed for a young population in a university academic context. Thus, Waterman et al. [12] designed an instrument composed of 21 items measuring a single factor, eudemonic well-being. For the validation process they used a sample of 1728 young people with a mean age of 20.04 years. They conducted a confirmatory factor analysis which showed acceptable fit indices for the single factor model. In addition, the reliability analysis showed an acceptable score of over 0.80. However, the validation process of an instrument is continuous, so the structures of questionnaires may vary. In this sense, Schutte, Wissing and Khumalo [17] aimed to analyze the factor structure of the questionnaire in university students (*N* = 325). They conducted three confirmatory factor analyses: the first one was based on one factor (eudaimonic well-being), the next one on three factors (sense of meaning and control, social competence and personal growth) and the last one on four factors (sense of meaning and purpose, sense of control and autonomy, social contribution and competence, personal growth and self-acceptance). Analyses showed acceptable fit rates only with the four-factor model. In the Spanish context, Waterman’s [12] Eudemonic Well-Being Questionnaire was validated by Salavera and Usan [18] in the context of secondary education. A total of 1031 young people participated in the validation process of the questionnaire. The results of their study revealed that the factor structure of the questionnaire consists of six factors (sense of control or autonomy, sense of meaning and purpose, personal expressiveness, sense of belonging, social contribution and competence, and personal growth and self-acceptance). In addition, the scale showed adequate reliability and concurrent validity.

Taking into account this background and the need for a questionnaire to analyze eudemonic well-being in athletes, the present study aims to adapt and validate the Questionnaire Eudemonic Well-Being by Salavera and Usan [18] for the sports context, analyzing its psychometric properties. In addition, in order to confirm the factorial structure of the questionnaire, the studies of Waterman [12] and Schutte, Wissing and Khumalo [17] will be taken into account. For this purpose, several factor analyses will be carried out, taking into account previous antecedents. In addition, an analysis of invariance with respect to gender, temporal stability and internal consistency will be carried out.

## 2. Method

### 2.1. Participants

The numbers of athletes who participated in the study were 2487 (1184 males and 1303 females) with a mean age of 26.43 years (SD = 4.69) (range: 17 years to 33 years), belonging to various first and second division sports clubs in Andalusia (Spain). The sample of participants was distributed as follows:

For the confirmatory factor analysis, 1788 athletes (856 males and 932 females) participated with a mean age of 26.27 years (SD = 4.78). Finally, for the temporal stability analysis, 699 athletes (328 men and 371 women) participated with a mean age of 27.66 years (SD = 4.08). They filled in the same questionnaire twice, two weeks apart.

### 2.2. Measurements

Questionnaire Eudemonic Well-Being. An adapted questionnaire from Salavera and Usan [18] was used, which in turn stems from Waterman’s version [12]. From its initial design, the questionnaire consists of 21 items that refer to elements of eudemonia. Responses to each of these items are made on a Likert scale ranging from 1 = strongly disagree to 6 = strongly agree).

### 2.3. Procedure

Before starting the validation process, it was necessary to adapt those items that made a generic mention of the life of adolescents and young people rather than the sporting life of athletes. For this process, several sports psychologists of reputed experience in the field of sport psychology and research were consulted to judge the suitability of the items [19], so as to ensure that the items obtained were well designed to measure the construct to be measured, without losing the original meaning.

Once the final version of the questionnaire was obtained, several sports clubs in Andalusia were contacted in order to request their collaboration in the present study. In this first contact, the aim of the study was explained to them and any doubts they had were answered. Those sports clubs that agreed to participate in the study allowed us to contact their athletes, who were also informed of the objective of the study and were also informed that their participation, if they so wished, would be voluntary and anonymous.

Those athletes who wished to participate in the study were required to give written informed consent.

The administration of the scale took place at the beginning of a mid-season training sessions, and was completed individually, with a member of the research group present to answer any questions. The time taken by the athletes to complete the entire questionnaire was around 12 min. On the other hand, those participants who took part in the temporal stability analysis completed the questionnaire twice at the beginning of the training session with a two-week gap between each intake.

## 3. Data Analysis

In order to analyze the psychometric properties of the Questionnaire Eudemonic Well-Being in a sport context, it was necessary to analyze its factor structure. Thus, several confirmatory factor analyses (CFA) were carried out in order to determine the factor structure of the questionnaire, taking into account the studies of Waterman [9] and Salavera and Usan [18] (six factors), Schutte, Wissing and Khumalo [17] (four and three factors) and Waterman et al. [12] (one factor). If the confirmatory factor analysis determines a multifactor structure of the questionnaire, a higher order factor analysis will be performed in order to group each of the factors into a higher factor that groups them together (see, [20]).

Once the factor structure was determined, multi-group analyses were carried out to analyze invariance with respect to gender, descriptive statistical analyses and the reliability of the instrument was tested through internal consistency analysis (Cronbach’s alpha) and an analysis of temporal stability (intra-class correlation index, ICC) by means of the test-retest. The statistical software used was SPSS (IBM, Armonk, NY, USA) and AMOS (IBM, Armonk, NY, USA), both in version 21.

For the CFA, a Bootstrapping of 6000 interactions was used together with the maximum likelihood method, since Mardia’s coefficient was very high (129.56). Despite this coefficient score, the estimators were not affected and were considered robust. Thus, the following fit indices (Table 1) [21,22] were taken into account to consider the model tested through the CFA as acceptable [21,22]:

## 4. Results

### 4.1. Confirmatory Factor Analysis

Four models were tested based on previous studies [8,12,18] revealing the following fit indices (Table 2).

**Figure 1:**
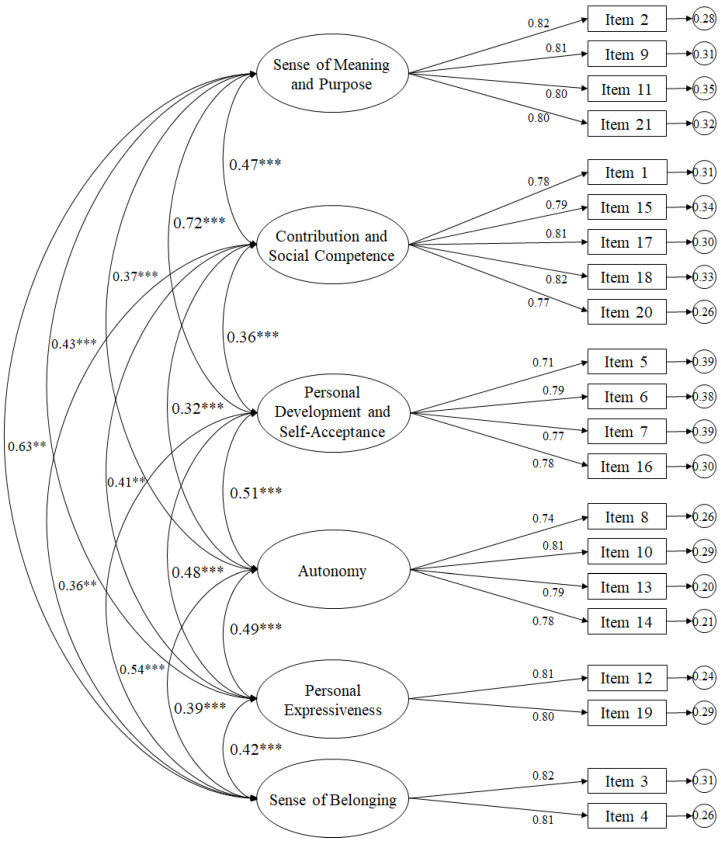
Diagram of the CFA belonging to the Questionnaire on Eudemonic Well-Being in a sport context. *** *p <* 0.001; ** *p <* 0.01.

Taking into account the fit indices described above, successive CFAs revealed the six-factor model to be acceptable. Therefore, the six-factor model (Figure 1) revealed through the CFA that the standardized regression weights ranged between 0.71 and 0.82 and were significant (*p* < 0.001). The correlations between the factors ranged between 0.35 and 0.72 and were also statistically significant (*p* < 0.001).

A CFA was also performed for a higher order model in order to group all the factors into a common index. The adjustment rates were adequate: χ^2^ (183. *N* = 1788) = 540.49, *p* < 0.001; χ^2^/df = 2.95; Tucker Lewis index (TLI) = 0.95; comparative fit index (CFI) = 0.95; incremental fit index (IFI) = 0.95; root mean square error of approximation (RMSEA) = 0.055; standardized root mean square residual (SRMSR) = 0.044.

### 4.2. Invariance Analysis by Gender

A multigroup analysis was conducted in order to test whether the factor structure of the questionnaire is not compromised by the gender variable (Table 3). For this purpose, several models are presented with successive restrictions. Thus, the absence of significant differences between Model 1 and Model 2 is a minimum criterion for accepting that the structure of the six-factor model and the higher-order model is invariant with respect to gender [23].

### 4.3. Descriptive Statistics, Reliability and Temporal Stability Analysis

Table 4 shows the clear reciprocity between the factors through Pearson’s correlation. In addition, the reliability analyses showed a score above 0.80. As for the temporal stability analysis, the intra-class correlation coefficients (ICC) and their confidence intervals (CI) were calculated, yielding a score above 0.80 for each of the factors.

## 5. Discussion

The adaptation and validation of the Eudemonic Well-being Scale of Salavera and Usan [18] to the sport context is the main objective of this study. The analyses have shown that the questionnaire constitutes an instrument that shows evidence of validity and reliability for both the six-factor model and the higher-order model, which will allow measuring the different aspects related to the eudemonic well-being of athletes in the sport context.

In this sense, the results shown in the CFA reflect that the factor structure of the questionnaire is robust for the six-factor model, which is in line with the version of Salavera and Usan [18] and with the theoretical proposal of Waterman [9]. However, it differs with the factor structure of Waterman’s [12] one-factor model and Schutte, Wissing and Khumalo’s [17] three- and four-factor model. The fit indices presented in this study show better fit indices for the CFA than previous studies [9,12,17,18]. In addition, the second CFA reflected that the factor structure of the higher-order model showed acceptable fit indices. These results cannot be compared with the preceding scales as they did not perform this type of analysis. This will allow researchers to group factors into a higher factor when the predictive model is overly complex [21,24]. These results show that the use of the scale can contribute to providing opportunities to understand how athletes develop their personal potential, from the philosophical perspective of eudemonia which is related to self-realisation and personal self-development [25,26]. In this sense, a study conducted with secondary school students in physical education showed that those students who achieved their academic goals experienced high eudemonia, which brought them happiness, increased their motivation and encouraged them to continue their personal growth [27].

On the other hand, the results have shown that the questionnaire is gender invariant, i.e., both men and women understand the questionnaire in a similar way. Likewise, the questionnaire shows adequate temporal stability so that participants’ responses in future studies will understand the items in a similar way over short periods of time.

In short, these results show that the Eudemonic Well-Being Scale towards the sport context shows evidence of reliability and validity, as well as being invariant with respect to gender. Although the questionnaire is statistically robust, there are a number of limitations to be taken into account. In this sense, the participants in this study were chosen at convenience, based on those sports clubs to which we had access and which wanted to participate in the study. In addition, this is a self-report questionnaire, so response bias may be affected. On the other hand, future studies should analyze the predictability of the questionnaire, i.e., whether the questionnaire measures what it should actually measure. For this purpose, a concurrent validity analysis can be carried out with respect to the original scale. Furthermore, future studies in the sport context should analyze the differences between hedonic and eudemonia and see their effects on athletes’ involvement.

## 6. Conclusions

This study shows that the eudemonic well-being questionnaire in the sport context is valid and reliable (Appendix A). Therefore, it can help to analyze the well-being of athletes in depth. In this sense, athletes are often under enormous pressure, which means that they are exposed to stressful situations and anxiety. Therefore, coaches and sports psychologists will be able to address other expedient lines of approach when dealing with alternative training methods and different communication techniques that contribute to improving the well-being of athletes.

## Figures and Tables

**Table 1:** Confirmatory factor analyses (CFA) adjustment indices.

Type Analysis	Adjustment Index
RMSEA	Equal or less than 0.06
SRMSR	Equal or less than 0.08
IFI	+0.95
TLI	+0.95
CFI	+0.95
χ^2^/degree freedom	Between 2 and 3

Note: RMSEA = root mean square error of approximation; SRMSR = standardized root mean square residual; IFI = incremental fit index; TLI = Tucker Lewis index; CFI = comparative fit index.

**Table 2:** Fit indices of the models after CFA.

Models	χ^2^	df	χ^2^/df	IFI	CFI	TLI	RMSEA	SRMSR
One-factor Model	649,10	189	3.43	0.92	0.92	0.91	0.64	0.53
3-factor Model	1269,26	186	6.82	0.69	0.69	0.69	1.06	0.78
4-factor Model	1058,53	183	5.78	0.89	0.89	0.89	0.72	0.69
6-factor Model	431,52	174	2.48	0.96	0.96	0.96	0.43	0.51

**Table 3:** Analysis of invariance by sex.

**Six-Factor Model**										
**Models**	**χ^2^**	*df*	**χ^2^/*df***	**Δχ^2^**	**Δ*df***	**CFI**	**TLI**	**IFI**	**RMSEA**	**SRMSR**
1	882.07	348	2.53	-	-	0.97	0.97	0.97	0.051	0.045
2	963.68	363	2.65	24.03	15	0.96	0.96	0.96	0.055	0.051
3	1106.65	405	2.73	160.16 **	57	0.96	0.96	0.96	0.061	0.055
4	1188.31	426	2.79	289.48 **	78	0.95	0.95	0.95	0.067	0.060
**Higher Order Model**										
**Models**	**χ^2^**	***df***	**χ^2^/*df***	**Δχ^2^**	**Δ*df***	**CFI**	**TLI**	**IFI**	**RMSEA**	**SRMSR**
1	945.43	366	2.58	-	-	0.96	0.96	0.96	0.052	0.046
2	1009.19	381	2.65	41.56	15	0.96	0.96	0.96	0.055	0.048
3	1079.20	402	2.68	97.76	36	0.95	0.95	0.95	0.060	0.050
4	1139.76	408	2.80	135.76 **	41	0.95	0.94	0.95	0.066	0.052
5	1187.66	414	2.87	142.47 **	48	0.94	0.94	0.94	0.071	0.056
6	1300.54	435	2.99	193.55 **	69	0.93	0.93	0.93	0.075	0.060

Note: ** *p* < 0.01. Six-factor model (Model 1 = unrestricted; Model 2 = invariance in measurement weights; Model 3 = structural-invariant-covariance; Model 4 = residual invariant measurement). Higher order model (Model 1 = unrestricted; Model 2 = invariance in measurement weights; Model 3 = invariant structural weights; Model 4 = invariant structural covariance; Model 5 = residual invariant structural; Model 6 = residual invariant measurement.

**Table 4:** Descriptive statistics, reliability and temporal stability analysis.

Factors	*M*	*SD*	α	AVE	1	2	3	4	5	6	ICC
1. Sense of Meaning and Purpose	3.23	0.70	0.88	0.66	-	0.38 **	0.59 ***	0.39 ***	0.58 **	0.55 **	0.88 (CI = 0.85–0.91)
2. Contribution and Social Competence	3.25	0.65	0.87	0.63		-	0.61 **	0.42 **	0.49 **	0.28 **	0.83 (CI = 0.80–0.88)
3. Personal Development and Self-Acceptance	3.56	0.85	0.85	0.60			-	0.47 **	0.68 **	0.51 **	0.81 (CI = 0.80–0.85)
4. Autonomy	3.78	1.02	0.84	0.62				-	0.27 **	0.30 **	0.85 (CI = 0.82–0.89)
5. Personal Expressiveness	3.33	0.49	0.81	0.65					-	0.58 **	0.88 (CI = 0.83–0.92)
6. Sense of Belonging	3.29	0.91	0.85	0.66						-	0.84 (CI = 0.82–0.87)

Note: ** *p* < 0.01; *** *p* < 0.001.

## Data Availability

Not applicable.

## Appendix A

Questionnaire of Eudemonic Well-Being in the sport context. This instrument was validated in Spanish.

**Sense of Meaning and Purpose**

2. Creo que descubrí quién soy realmente, como deportista (I think I discovered who I really am as an athlete).

9. Puedo decir que he encontrado mi meta deportiva (I can say that I have found my sporting goal).

11. Hasta ahora, no he descubierto qué hacer con mi vida deportiva (So far, I haven’t figured out what to do with my sporting life).

21. Como deportista, creo que sé lo que debía hacer en la vida (As an athlete, I think I know what I was meant to do in life).

**Contribution and Social Competence**

1. Como deportista, me involucro intensamente en los entrenamientos (As an athlete, I am heavily involved in training).

15. Cuando participo en actividades deportivas que involucran mis mejores cualidades, tengo una gran sensación personal (When I participate in sporting activities that involve my best qualities, I have a great personal feeling).

17. Encuentro que muchas de las cosas que practico durante los entrenamientos son personalmente importantes para mí (I find that many of the things I practice during training sessions are personally important to me).

18. Es importante para mí sentirme realizado/a por la actividad deportiva que realize (It is important for me to feel fulfilled by the sporting activity I do).

20. Como deportista, encuentro difícil involucrarme en las cosas que hago (As an athlete, I find it difficult to get involved in the things I do).

**Personal Development and Self-Acceptance**

5. Es más importante disfrutar de las cosas que hago cuando hago deporte, que el impresionar a los demás por ello (It is more important to enjoy the things I do when I do sport, than to impress others about it).

6. Creo que sé cuáles son mis mejores cualidades como deportista y trato de desarrollarlas siempre que sea possible (I believe that I know what my best qualities are as an athlete and I try to develop them whenever possible).

7. Por lo general, las personas a mi alrededor saben mejor que yo mismo, lo que sería bueno para mí (People around me usually know better than I do what would be good for me).

16. Me siento confundido si son realmente buenas mis habilidades y capacidades (I feel confused whether my skills and abilities are really good).

**Autonomy**

8. Me siento mejor como deportista, cuando estoy haciendo algo en lo que vale la pena invertir una gran cantidad de esfuerzo (I feel best as an athlete when I am doing something that is worth a great deal of effort).

10. Como deportista, si no encuentro gratificante lo que estoy haciendo, no creería poder seguir haciéndolo (As an athlete, if I don’t find what I am doing rewarding, I wouldn’t believe I could continue doing it).

13. Creo que es importante saber que lo que estoy haciendo encaja con los propósitos deportivos que vale la pena perseguir (I think it is important to know that what I am doing fits in with the sporting purposes that are worth pursuing).

14. Por lo general, sé lo que debo hacer porque algunas acciones me parecen correctas (I usually know what I should do because some actions seem right to me).

**Personal Expressiveness**

3. Creo que como deportista sería ideal si me salieran fácilmente las habilidades que pretendo aprender (I believe that as an athlete I would be ideal if the skills I intend to learn came easily to me).

4. Mis creencias como deportista dan sentido a mi actividad deportiva (My beliefs as an athlete give meaning to my sporting activity and my beliefs as an athlete give meaning to my sporting activity).

**Sense of Belonging**

12. No puedo entender por qué algunos de mis compañeros quieren trabajan tanto durante los entrenamientos/competición (I can’t understand why some of my teammates want to work so hard during training/competition).

19. Si algo es realmente difícil, probablemente no valga la pena luchar por ello (If something is really difficult, it is probably not worth fighting for).
